# Supplementary material for: Computational Model for Human 3D Shape Perception From a Single Specular Image
Source: Front Comput Neurosci. 2019 Mar 1;13:10. doi: 10.3389/fncom.2019.00010 (PMC6407488; doi:10.3389/fncom.2019.00010)
Supplement: Supplementary file 1 [file Presentation_1.pdf]

Appendix A: Additional explanation of Figure 2

In this case, surface orientation θ_s is close to zero and satisfies $\cos^2\theta_s \geq (1-\alpha_s)\sin^2\theta_s$. Therefore, the surface second derivative sign of vertical orientation $\mathrm{sgn}\left(-\frac{\partial^2 z}{\partial y^2}\right)$ equals the sign of large surface second derivative σ_{max} (see Materials and Methods). Combined with Equation (5), the vertical polarity corresponds to σ_{max}. Note that the vertical polarity has error due to the boundary effect and the slight deviation from the light from above assumption. In the case of $\theta_s \approx 90°$, σ_{min} is relevant to the vertical polarity. Whether σ_{max} or σ_{min} corresponds to the vertical polarity depends on surface orientation θ_s.

Appendix B: Psychophysical experiment results

Five subjects were first asked whether the local 3D surface around the red crosses in Figures 6A,B looks convex or concave. After that, they were asked whether the true 3D shape (Figure 6C) or the recovered 3D shape (Figure 6D or Figure 6E) was more similar to the perceived 3D shape from the image. Four of five subjects answered that the local surface of Figure 6A looked concave and only one thought that it looked convex. All five subjects answered that the local surface of Figure 6B looked convex. Four of five subjects answered that the recovered shape (Figure 6D) was closer to the perceived shape of the image shown in Figure 6A, and one thought that the true shape (Figure 6C) was closer. Four of five subjects answered that the recovered shape (Figure 6E) was closer to the perceived shape from the image shown in Figure 6B and one answered that the true shape was closer.

Appendix C: Minimization of cost function

We optimized $\boldsymbol{\sigma}_{max}$, $\boldsymbol{\sigma}_{min}$, and $\mathbf{k}_a$ by minimizing cost function E (Equation 18) as follows. First, we optimized the second derivative signs, $\boldsymbol{\sigma}_{max}$ and $\boldsymbol{\sigma}_{min}$, assuming that the second derivative magnitude is constant as $\mathbf{k}_a=\mathbf{1}$. After that, we optimized second derivative magnitude $\mathbf{k}_a$. We first optimized the signs for the following reasons. First, the signs are more easily optimized because the vertical polarity gives reasonable initial values. Second, the influence of the sign error on the magnitude optimization is larger than the opposite case because the magnitude amplifies the sign effect.

Mean field algorithm for optimizing the signs

With the assumption that $\mathbf{k}_a=\mathbf{1}$, the cost function becomes a simple quadratic form of the summarized signs $\boldsymbol{\sigma}_{all} = (\boldsymbol{\sigma}_{max}^T, \boldsymbol{\sigma}_{min}^T)^T$:

$$E = -\frac{1}{2}\boldsymbol{\sigma}_{\mathrm{all}}^T\mathbf{J}\boldsymbol{\sigma}_{\mathrm{all}} - \mathbf{h}_{\mathrm{all}}^T\boldsymbol{\sigma}_{\mathrm{all}} + \mathrm{const.}, \tag{A1}$$

where $\mathbf{J} = (\mathbf{D}_{vv}^T, \mathbf{D}_{uu}^T\mathbf{H})^T\mathbf{A}^{-1}(\mathbf{D}_{vv}^T, \mathbf{D}_{uu}^T\mathbf{H})$ and $\mathbf{h}_{\mathrm{all}} = (\mathbf{h}_{\mathrm{max}}^T, \mathbf{h}_{\mathrm{min}}^T)^T$.

Equation (A1) is a hard optimization problem because $\boldsymbol{\sigma}_{all}$ are binary parameters. However, we can utilize an existing approximation method since function Equation (A1) is the same as the Ising model. We used a naive mean field approximation to derive the optimization algorithm (Parisi, 1988).

1. Initial value: The initial values are determined by the vertical polarity (Equation 7) and set to zero where there is no information:

$$\sigma_{\max}(x,y) = \begin{cases} p_v(x,y) & (x,y \in \Omega_a) \\ 0 & (x,y \in \Omega_b) \end{cases}, \tag{A2}$$

$$\sigma_{\min}(x,y) = \begin{cases} 0 & (x,y \in \Omega_a) \\ p_v(x,y) & (x,y \in \Omega_b) \end{cases}. \tag{A3}$$

Additionally, the initial values near the boundary are determined so that $\sigma_{\max} = +1$ and $\sigma_{\min}$ equal the curvature sign of the image contour (Supplementary Figure 5).

2. Iteration: This update equation is repeated N_σ times (N_σ =100 in this study):

$$\boldsymbol{\sigma}_{\text{all}} = \tanh\{\beta(\mathbf{J}\boldsymbol{\sigma}_{\text{all}} + \mathbf{h}_{\text{all}})\}, \tag{A4}$$

where β is a constant representing the inverse temperature of the system. If β is either small or large, the fluctuation is either large or small. To search for the global minimum, simulated annealing was used in this study: the initial value of the inverse temperature is $\beta = \beta_0 / |\mathbf{J}|$ and increased per iteration as $\beta = 1.1\beta$, where $\beta_0 = 10$, and $|\mathbf{J}|$ is the maximum eigenvalue of matrix **J**.

3. Result: The solution is obtained by extracting the sign:

$$\boldsymbol{\sigma}_{\text{all}} = \text{sgn}(\boldsymbol{\sigma}_{\text{all}}). \tag{A5}$$

Optimization of second derivative amplitudes

Given $\boldsymbol{\sigma}_{\max}$ and $\boldsymbol{\sigma}_{\min}$, the cost function becomes a simple quadratic form of second derivative magnitude $\mathbf{k}_a$:

$$E = \frac{1}{2}\mathbf{k}_a^T\mathbf{R}\mathbf{k}_a + \text{const.}, \tag{A6}$$

where $\mathbf{R} = \mathbf{I} + \mathbf{H}^2 - (\mathbf{D}_{vv}^T\boldsymbol{\Sigma}_{\max} + \mathbf{D}_{uu}^T\mathbf{H}\boldsymbol{\Sigma}_{\min})^T\mathbf{A}^{-1}(\mathbf{D}_{vv}^T\boldsymbol{\Sigma}_{\max} + \mathbf{D}_{uu}^T\mathbf{H}\boldsymbol{\Sigma}_{\min})$; $\boldsymbol{\Sigma}_{\max}$ and $\boldsymbol{\Sigma}_{\min}$ are diagonal matrices with diagonal elements $\boldsymbol{\sigma}_{\max}$ and $\boldsymbol{\sigma}_{\min}$. In the minimization of the cost function, a constraint, where the mean value of the second derivative magnitude is 1 as $\mathbf{k}_a^T\mathbf{1}/N_\Omega = 1$, is introduced to avoid a trivial solution, $\mathbf{k}_a = \mathbf{0}$. The solution is obtained by the Lagrange multiplier:

$$L = \frac{1}{2}\mathbf{k}_a^T\mathbf{R}\mathbf{k}_a - \lambda\left(\frac{1}{N_\Omega}\mathbf{k}_a^T\mathbf{1} - 1\right). \tag{A7}$$

Again, the sign optimization precedes the magnitude optimization. Although $k_a \geq 0$ by definition, the cost function is minimized by removing this restriction and the solution is given by $\frac{\partial L}{\partial \mathbf{k}_a} = \mathbf{0}$ and $\frac{\partial L}{\partial \lambda} = 0$:

$$\mathbf{k}_a = \frac{N_\Omega}{(\mathbf{1}^T \mathbf{R}^{-1} \mathbf{1})} \mathbf{R}^{-1} \mathbf{1}. \tag{A8}$$

Then, based on Equations (3) and (4), we flip the signs as $k_a(x,y) = -k_a(x,y)$, $\sigma_{max}(x,y) = -\sigma_{max}(x,y)$, and $\sigma_{min}(x,y) = -\sigma_{min}(x,y)$ where $k_a(x,y)<0$. This procedure minimizes the cost function greater than the magnitude optimization under constraint $k_a \geq 0$ by simultaneously optimizing the signs and the magnitude. For the sign optimization, we repeat this procedure N_k times (N_k=10 in this study) by updating σ_{max} and σ_{min} and alternately repeat the mean field algorithm described above and this procedure N_{loop} times (N_{loop}=10 in this study). Note that, in the mean field algorithm, the assumption that $\mathbf{k}_a=\mathbf{1}$ holds during this loop.

After sufficient optimization of the second derivative signs, the optimized magnitude is given by Equation (A8).

Appendix D: Minimization of second cost function

The improved solution is obtained by second cost function C' (Equation 12). The second cost function is complicated, which makes the global search based on it difficult. Therefore, we utilize the second cost function to locally improve the solution obtained by the first cost minimization. The second cost function is represented without z by the same procedure of Equations (17) and (18):

$$E' = -\frac{1}{2}(\mathbf{D}_{vv}^T \mathbf{K}_a^{-1} \boldsymbol{\sigma}_{\max} + \mathbf{D}_{uu}^T \mathbf{H} \mathbf{K}_a^{-1} \boldsymbol{\sigma}_{\min})^T \mathbf{A}'^{-1} (\mathbf{D}_{vv}^T \mathbf{K}_a^{-1} \boldsymbol{\sigma}_{\max} + \mathbf{D}_{uu}^T \mathbf{H} \mathbf{K}_a^{-1} \boldsymbol{\sigma}_{\min}) + \frac{1}{2} \mathbf{1}^T (\mathbf{I} + \mathbf{H}^2) \mathbf{1} - (\mathbf{h}_{\max}^T \boldsymbol{\sigma}_{\max} + \mathbf{h}_{\min}^T \boldsymbol{\sigma}_{\min}), \tag{A9}$$

where $\mathbf{A}' = \mathbf{D}_{vv}^T \mathbf{K}_a^{-2} \mathbf{D}_{vv} + \mathbf{D}_{uu}^T \mathbf{K}_a^{-2} \mathbf{D}_{uu} + 2\mathbf{D}_{uv}^T \mathbf{K}_a^{-2} \mathbf{D}_{uv} + \mathbf{B}$. Cost function E' is minimized by repeating the mean field algorithm and the gradient descent. We used the optimized σ_{max} and σ_{min} by the first cost function as initial values. However, the initial value of the second derivative magnitude was $k_a = 1$, because the resultant cost function was minimized greater than the case where we used the optimized k_a by the first cost function as initial values.

Mean field algorithm for optimizing the signs

Cost function E' is a simple quadratic form of the summarized signs $\boldsymbol{\sigma}_{all} = (\boldsymbol{\sigma}_{max}^T, \boldsymbol{\sigma}_{min}^T)^T$:

$$E' = -\frac{1}{2} \boldsymbol{\sigma}_{\text{all}}^T \mathbf{J}' \boldsymbol{\sigma}_{\text{all}} - \mathbf{h}_{\text{all}}^T \boldsymbol{\sigma}_{\text{all}} + \text{const.}, \tag{A10}$$

where $\mathbf{J}' = (\mathbf{D}_{vv}^T \mathbf{K}_a^{-1}, \mathbf{D}_{uu}^T \mathbf{H} \mathbf{K}_a^{-1})^T \mathbf{A}'^{-1} (\mathbf{D}_{vv}^T \mathbf{K}_a^{-1}, \mathbf{D}_{uu}^T \mathbf{H} \mathbf{K}_a^{-1})$. The mean field algorithm (Equations (A4) and (A5)) optimized $\boldsymbol{\sigma}_{all}$.

Optimization of second derivative amplitudes

Since cost function E' is complicated with respect to $\mathbf{k}_a$, the gradient descent was used to optimize $\mathbf{k}_a$:

$$\mathbf{k}_a^{(i+1)} = \mathbf{k}_a^{(i)} - \gamma \nabla E'\left(\mathbf{k}_a^{(i)}\right), \tag{A11}$$

where $\gamma = 0.1$ in this study. We repeated this iteration 100 times. Furthermore, we alternately repeated the mean field algorithm and the gradient descent 10 times.

Effect of second cost minimization on results

We first obtained intermediate solutions based on the first cost minimization (Equation 11) and improved them based on the second cost minimization (Equation 12). Here, we show the difference of the results. The recovered shapes of the glossy condition by the first cost minimization are shown in Supplementary Figure 6. The recovered shape looks too smoothed and less bumpy than the improved solutions shown in Figure 4. The average values of the global and the local interior depth correlation for the 12 objects were $r_g = 0.82$ and $r_{li} = 0.71$. These depth correlation values were slightly worse than the improved solutions. The small difference is due to the fact that the estimated signs of the two conditions were almost the same (the agreement rates of σ_{max} and σ_{min} were 0.996 and 0.94) indicating that the surface second derivative signs were not improved by the second cost minimization because the solutions by the gradient descent are easily trapped into the local minimum. We interpreted why the appearance of the recovered shapes and the depth correlation values was improved by the second cost minimization as follows. The first cost function depends strongly on the shapes near the boundary because the cost is proportional to second derivative magnitude k_a (Equation 10), and the second derivative magnitude is generally large near the boundary. As a result, the recovered shapes are insensitive to bumps far from the boundary. In contrast, because the second cost function is normalized by second derivative magnitude k_a, the shapes were recovered with equal weighing to the inner region as well as near the boundary.
