# Supplementary material for: Computational Model for Human 3D Shape Perception From a Single Specular Image
Source: Front Comput Neurosci. 2019 Mar 1;13:10. doi: 10.3389/fncom.2019.00010 (PMC6407488; doi:10.3389/fncom.2019.00010)
Supplement: Supplementary file 2 [file Data_Sheet_1.PDF]

Supplementary Material

Computational Model for Human 3D Shape Perception from a Single Specular Image

Takeaki Shimokawa*, Akiko Nishio, Masa-aki Sato, Mitsuo Kawato, Hidehiko Komatsu

*** Correspondence:** Takeaki Shimokawa: shimokawa@atr.jp

Supplementary Note 1: Detailed analysis of proposed algorithm

We examined the effects of the peripheral conditions on the shape recovery.

Shape recovery without contour curvature signs

Our proposed algorithm obtains the surface second derivative signs near the boundary from the 2D contour sign: σ_{max} is +1, and σ_{min} equals the contour's curvature sign (Koenderink, 1984), which is shown in Supplementary Figure 5. These signs are utilized as the initial values of σ_{max} and σ_{min} and incorporated in the cost function (see the 'Formulation of cost function' of the Materials and Methods section).

To check the necessity of this boundary constraint, the shapes were recovered from the glossy surfaces without it. As a result, the estimation performances of objects #9 and #12 degraded significantly. The global depth correlation of #9 degraded from 0.89 to 0.07; the global and local interior depth correlations of #12 degraded from 0.80 to -0.01 and 0.60 to 0.25. However, the estimation performances of the other 10 objects were not changed. The average and standard deviation of the global and local interior depth correlation for the ten objects between the recovered shapes with and without this boundary constraint were 0.99 ± 0.02 and 0.98 ± 0.06. This result indicates that this boundary constraint affects a limited number of objects depending on their shapes.

Shape recovery under another illumination

To examine the robustness of the proposed algorithm, shapes were recovered from the glossy surfaces rendered under another illumination environment (Campus at Sunset of the Devebec dataset). In this condition, the average values of the mean absolute error of the orientation and the anisotropy for the 12 objects were 12.9° and 0.16. The average values of the correct ratio of the initial values of σ_{max} and σ_{min} for the 12 objects were 0.73 and 0.66. The errors of the image cues were larger than those of the glossy condition. The average values of the global and the local interior depth correlation for the 12 objects were $r_g = 0.76$ and $r_{li} = 0.63$. The average values of the correct ratio of the estimated σ_{max} and σ_{min} for the 12 objects were 0.81 and 0.67. The reason why the estimation performances were lower than the glossy condition is presumably due to the large orientation errors and the large initial second derivative sign errors. The mismatch between the light

from above prior and the Sunset illumination environment might also negatively affect the initial second derivative signs and the recovered shapes.

To further validate our algorithm, we recovered shapes from glossy surfaces under other illumination environments in the Devebec dataset. These environments consist of seven human-made environments (Grace Cathedral, St. Peter's Basilica, the Uffizi Gallery, Galileo's Tomb, Kitchen, and Overcast Breezeway) and one natural environment at a sunset condition (Funston Beach at Sunset). The following are the errors of image cues and estimation performances averaged for these eight environments and 12 objects: the mean absolute errors of orientation and anisotropy were 15.0° and 0.18; the correct ratios of the initial values of σ_{max} and σ_{min} were 0.68 and 0.63; the global and local interior depth correlations were $r_g = 0.74$ and $r_{li} = 0.61$; the correct ratios of the estimated σ_{max} and σ_{min} were 0.78 and 0.67. The results show the algorithm’s robustness, although the estimation performances under these illumination environments were lower than those under the natural illumination environment (Eucalyptus Grove).

Image resolution

To investigate how fine image resolution is desirable for the shape recovery, the shapes were recovered from the glossy surfaces with various image sizes. The average values of (r_g, r_{li}, correct ratio of estimated σ_{max}, and correct ratio of estimated σ_{min}) for the 12 objects of image size of 256 × 256, 512 × 512, 2048 × 2048, 4096 × 4096 were (0.75, 0.73, 0.82, 0.68), (0.82, 0.73, 0.86, 0.71), (0.83, 0.72, 0.87, 0.72), and (0.82, 0.72, 0.86, 0.71), respectively. The results of the image size of 1024 × 1024 are shown in Table 4. The result indicates that an image size of 512 × 512 is adequate for the proposed shape recovery algorithm.

Supplementary Note 2: Depth correlations without slant calibration

Here, we summarize the depth correlation values of all four conditions when we simply calculated the correlation of the estimated surface depth and the true surface depth without the affine transformation to calibrate the slant. The overall trend did not change with or without the slant calibration.

In the glossy condition, the global depth correlations of 12 objects (#1, #2,. . . #12) were r_g = 0.95, 0.88, 0.85, 0.88, 0.93, 0.84, 0.88, 0.94, 0.79, 0.62, 0.19, 0.54 (average r_g = 0.77), and the local interior depth correlations of 12 objects (#1, #2,. . . #12) were r_{li} = 0.96, 0.78, 0.58, 0.76, 0.95, 0.87, 0.75, 0.94, -, 0.35, -, 0.51 (average r_{li} = 0.75).

In the mirrored condition, the global depth correlations were r_g = 0.91, 0.87, 0.74, 0.86, 0.92, 0.87, 0.82, 0.92, 0.79, 0.64, 0.19, 0.39 (average r_g = 0.74), and the local interior depth correlations were r_{li} = 0.94, 0.78, 0.48, 0.77, 0.95, 0.89, 0.62, 0.89, -, 0.50, -, 0.46 (average r_{li} = 0.73).

In the noLFAP condition, the global depth correlations were r_g = 0.92, 0.82, 0.78, 0.79, 0.89, 0.85, 0.79, 0.95, 0.79, 0.46, 0.23, 0.23 (average r_g = 0.71), and the local interior depth correlations were r_{li} = 0.94, 0.50, 0.27, 0.40, 0.83, 0.63, 0.50, 0.95, -, -0.03, -, -0.02 (average r_{li} = 0.50).

In the shapeOF condition, the global depth correlations were r_g = 0.97, 0.86, 0.93, 0.94, 0.94, 0.89, 0.94, 0.97, 0.28, 0.68, 0.46, 0.36 (average r_g = 0.77), and the local interior depth correlations were r_{li} = 0.98, 0.86, 0.91, 0.90, 0.99, 0.95, 0.90, 0.99, -, 0.42, -, 0.67 (average r_{li} = 0.86).

Supplementary References

Koenderink, J. J. (1984). What does the occluding contour tell us about solid shape? *Perception* 13, 321–330.

Supplementary Figures

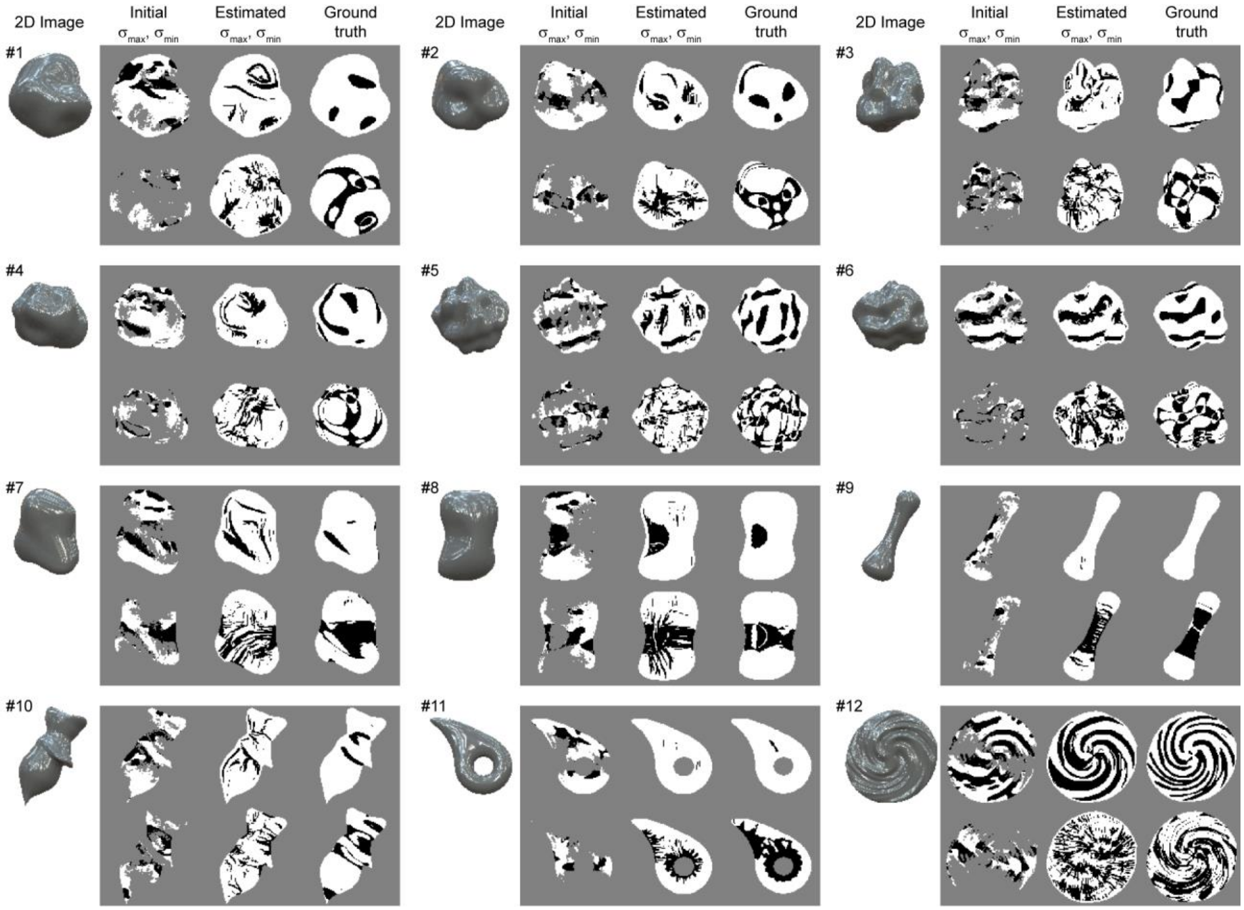

Supplementary Figure 1. Initial and estimated σ_{max} and σ_{min} from glossy surfaces. Initial, estimated and ground-truth signs of surface second derivatives are represented in gray scale. White represents positive and black represents negative. Results show that vertical polarity gives reasonable initial values, and optimization improves them and resolves remaining ambiguity.

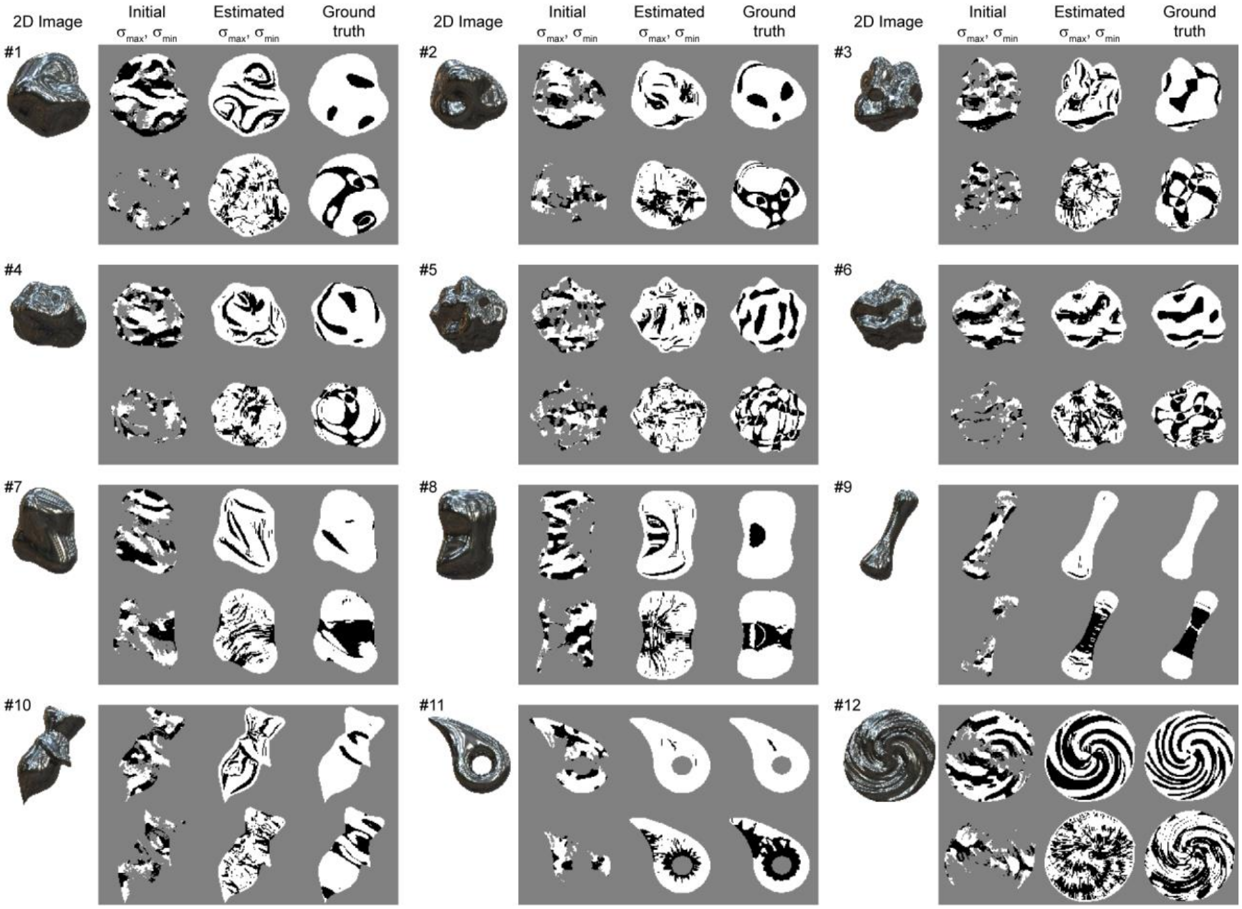

Supplementary Figure 2. Initial and estimated σ_{max} and σ_{min} from mirrored surfaces. Initial, estimated, and ground-truth signs of surface second derivatives are represented in gray scale. White represents positive and black represents negative. Results show that initial values are worse than those of glossy surfaces and thus estimated values are noisier than glossy surfaces.

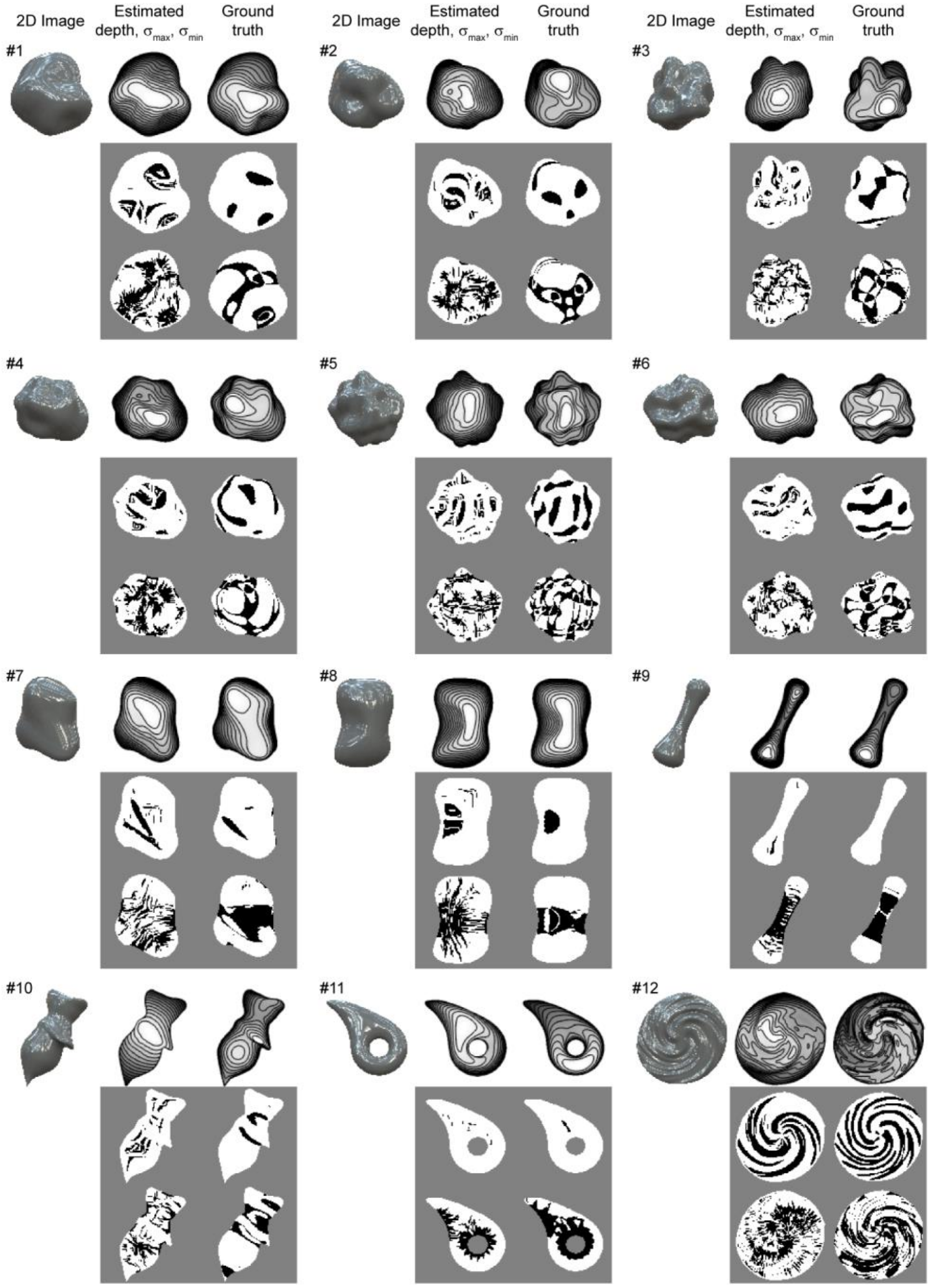

Supplementary Figure 3. Recovered 3D shapes and estimated σ_{max} and σ_{min} in noLFAP condition. Recovered surface shapes and ground-truth shapes are represented by depth map and contour lines. Estimated and ground-truth signs of surface second derivatives are represented in gray scale. Estimation performance of noLFAP condition was lowest among four conditions. Each object's values of estimation performance are summarized in Supplementary Table 1.

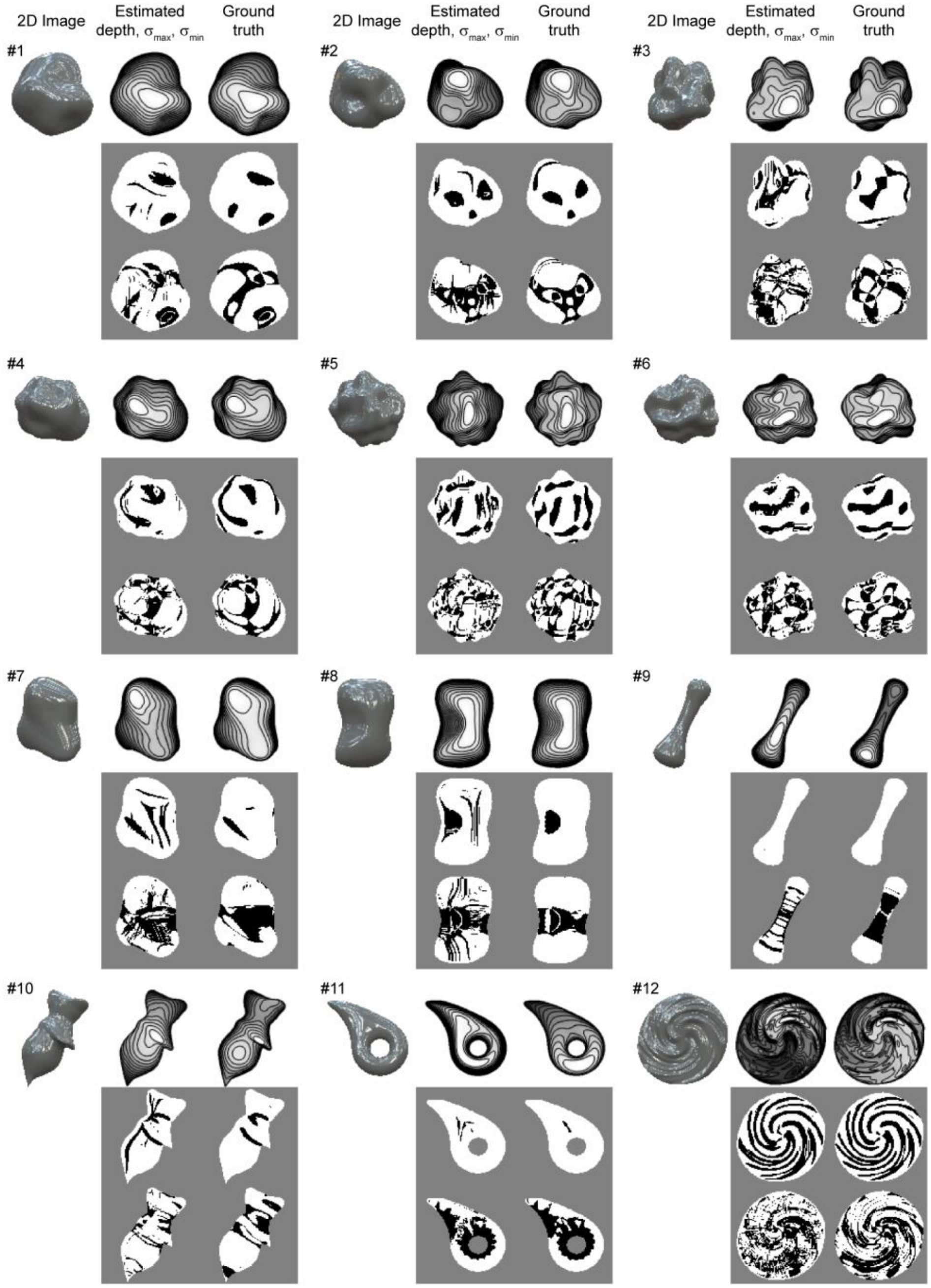

Supplementary Figure 4. Recovered 3D shapes and estimated σ_{max} and σ_{min} in shapeOF condition. Recovered surface shapes and ground-truth shapes are represented by depth map and contour lines. Estimated and ground-truth signs of surface second derivatives are represented in gray scale. Estimation performance of shapeOF condition was highest among four conditions. Each object's values of estimation performance are summarized in Supplementary Table 2.

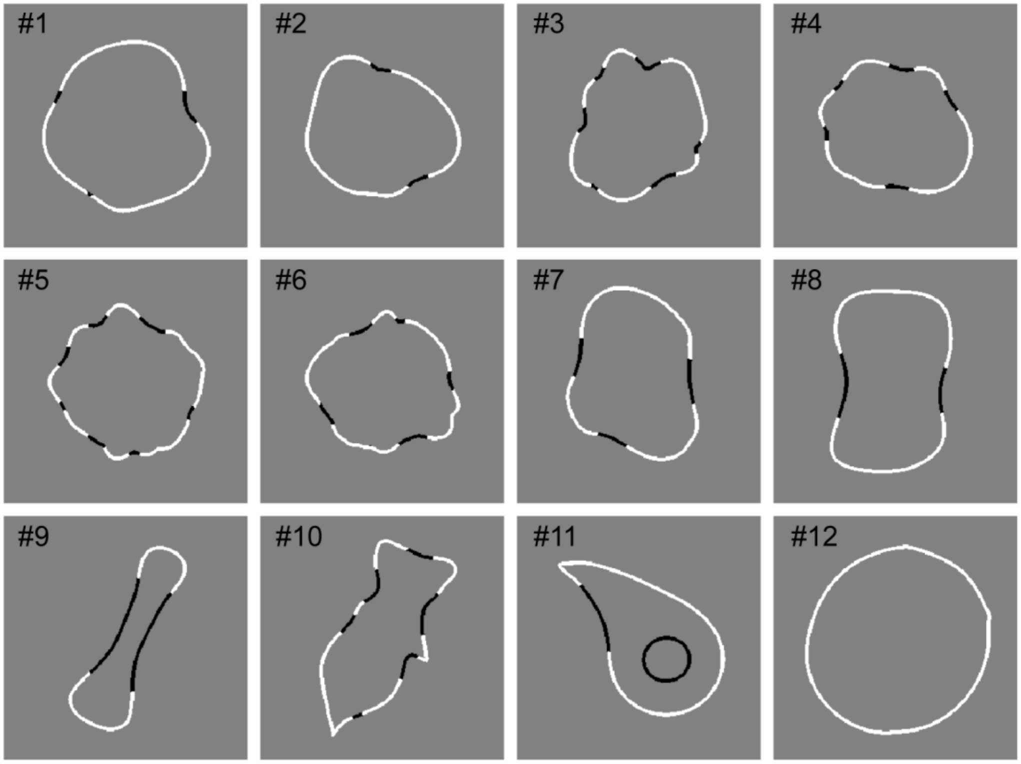

Supplementary Figure 5. Curvature sign of contour. White represents positive and black represents negative.

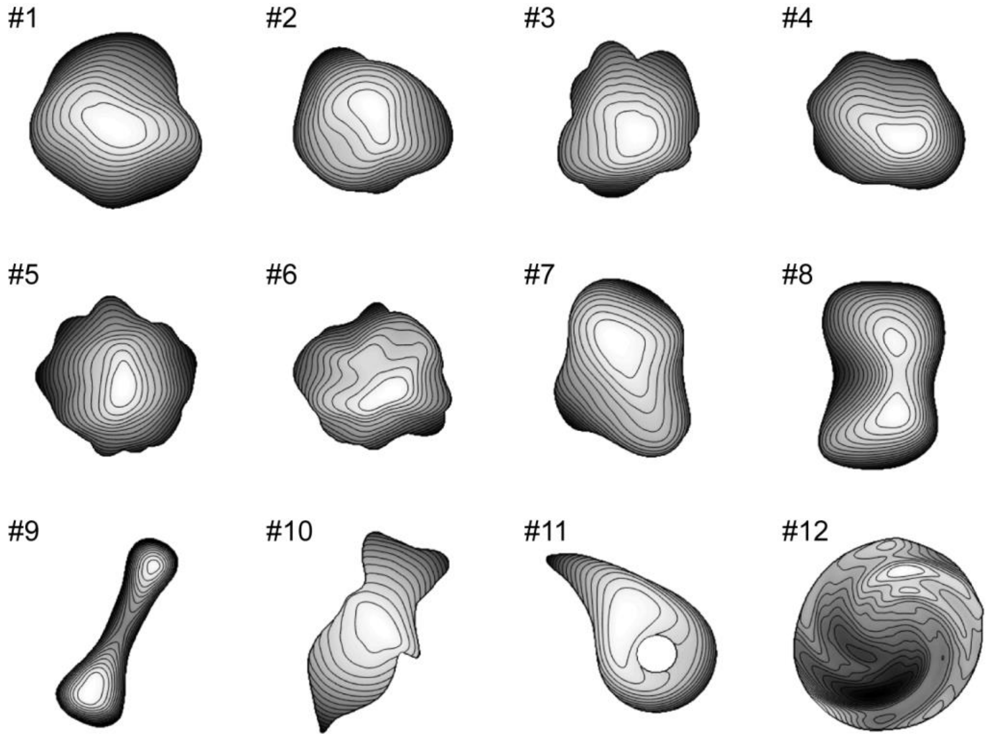

Supplementary Figure 6. Intermediate solutions of 3D shape (glossy condition). Recovered surface shapes by first cost minimization are represented by depth map and contour lines. Shape recovery performances of 12 objects (#1, #2,. . . #12) were as follows: global depth correlation r_g = 0.97, 0.85, 0.85, 0.78, 0.91, 0.94, 0.88, 0.96, 0.90, 0.61, 0.66, 0.51 (average r_g = 0.82); local interior depth correlation r_{li} = 0.96, 0.56, 0.62, 0.54, 0.91, 0.91, 0.80, 0.94, -, 0.34, -, 0.52 (average r_{li} = 0.71).

Supplementary Tables

Supplementary Table 1. Estimation performance of each object in noLFAP condition.

	#1	#2	#3	#4	#5	#6	#7	#8	#9	#10	#11	#12	average
r_g	0.94	0.83	0.79	0.73	0.87	0.90	0.79	0.95	0.93	0.51	0.70	0.31	0.77
r_{li}	0.96	0.35	0.33	0.29	0.79	0.69	0.61	0.97	-	0.15	-	0.05	0.52
σ_{max}	0.83	0.79	0.74	0.69	0.68	0.70	0.87	0.92	0.97	0.81	0.98	0.59	0.80
σ_{min}	0.73	0.62	0.60	0.58	0.56	0.63	0.66	0.77	0.82	0.65	0.81	0.57	0.67

Global and local interior depth correlations of recovered shapes and correct ratios of estimated signs of surface second derivative.

Supplementary Table 2. Estimation performance of each object in shapeOF condition.

	#1	#2	#3	#4	#5	#6	#7	#8	#9	#10	#11	#12	average
r_g	0.99	0.99	0.94	0.92	0.94	0.92	0.94	0.98	0.25	0.72	0.93	0.91	0.87
r_{li}	0.98	0.97	0.94	0.84	0.96	0.93	0.87	0.99	-	0.47	-	0.89	0.88
σ_{max}	0.95	0.93	0.85	0.88	0.90	0.92	0.89	0.91	1.00	0.90	0.97	0.95	0.92
σ_{min}	0.86	0.84	0.71	0.82	0.79	0.81	0.79	0.81	0.66	0.81	0.91	0.79	0.80

Global and local interior depth correlations of recovered shapes and correct ratios of estimated signs of surface second derivative.
